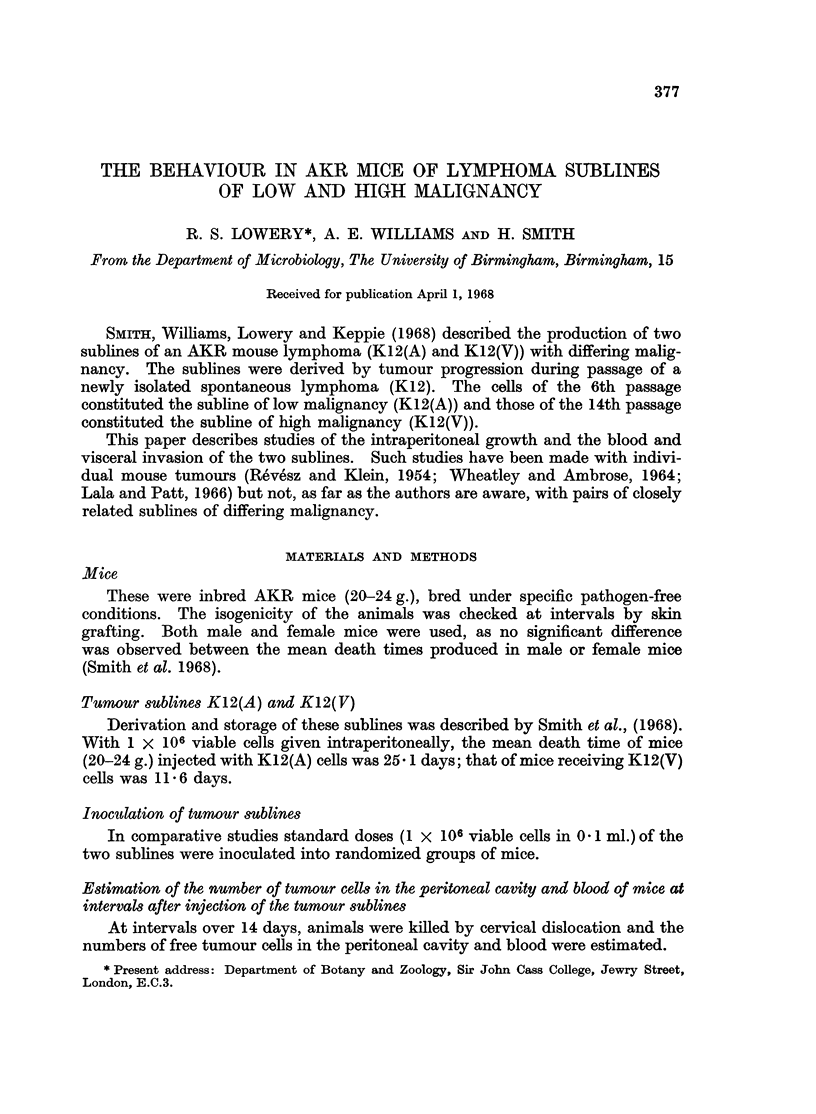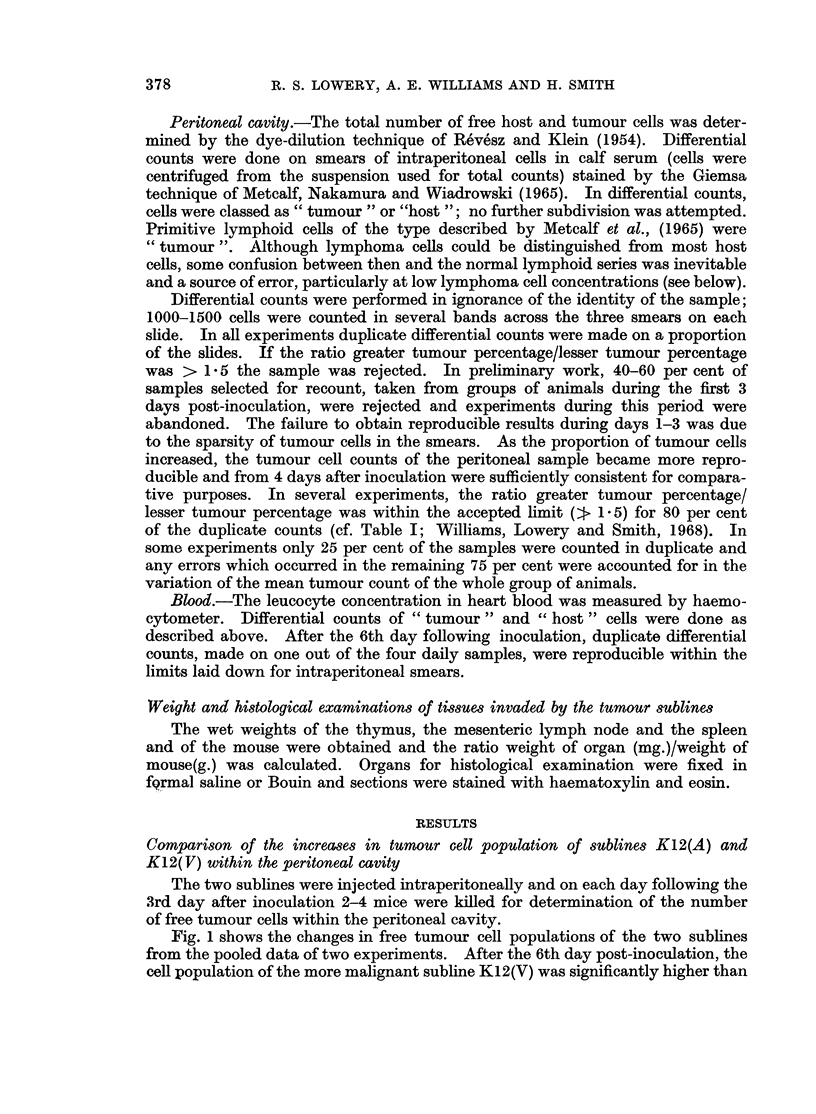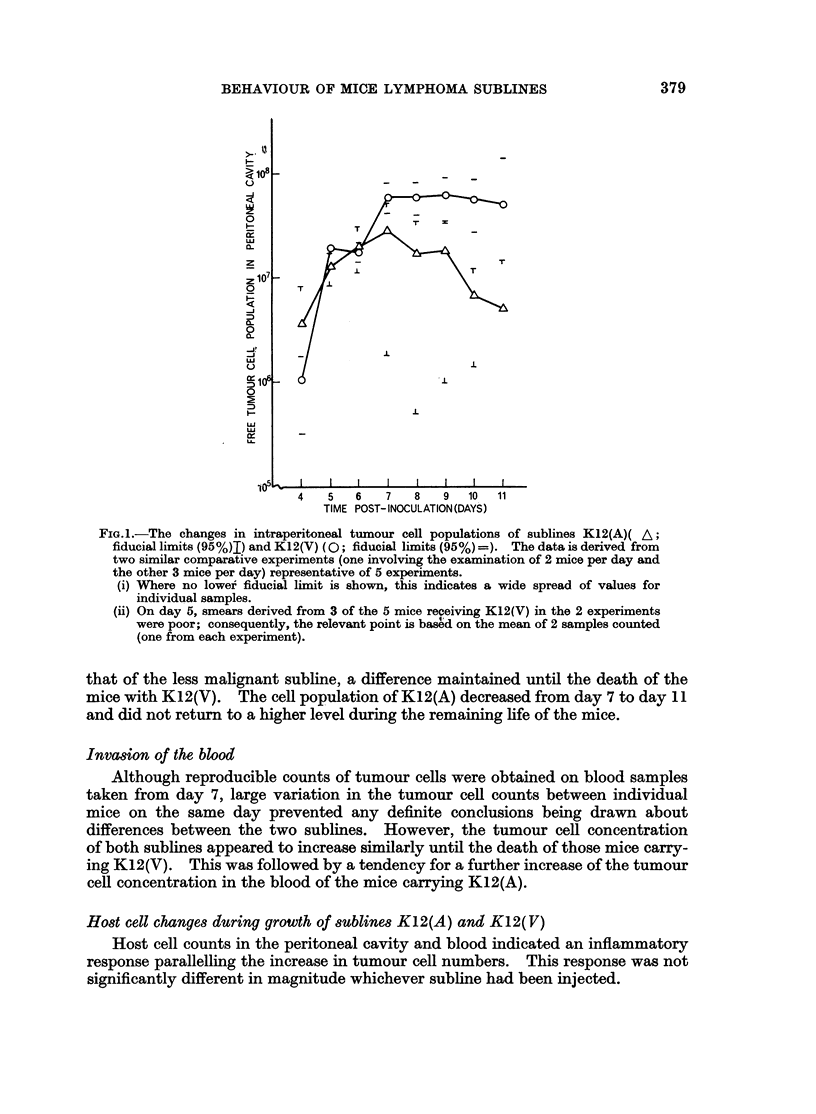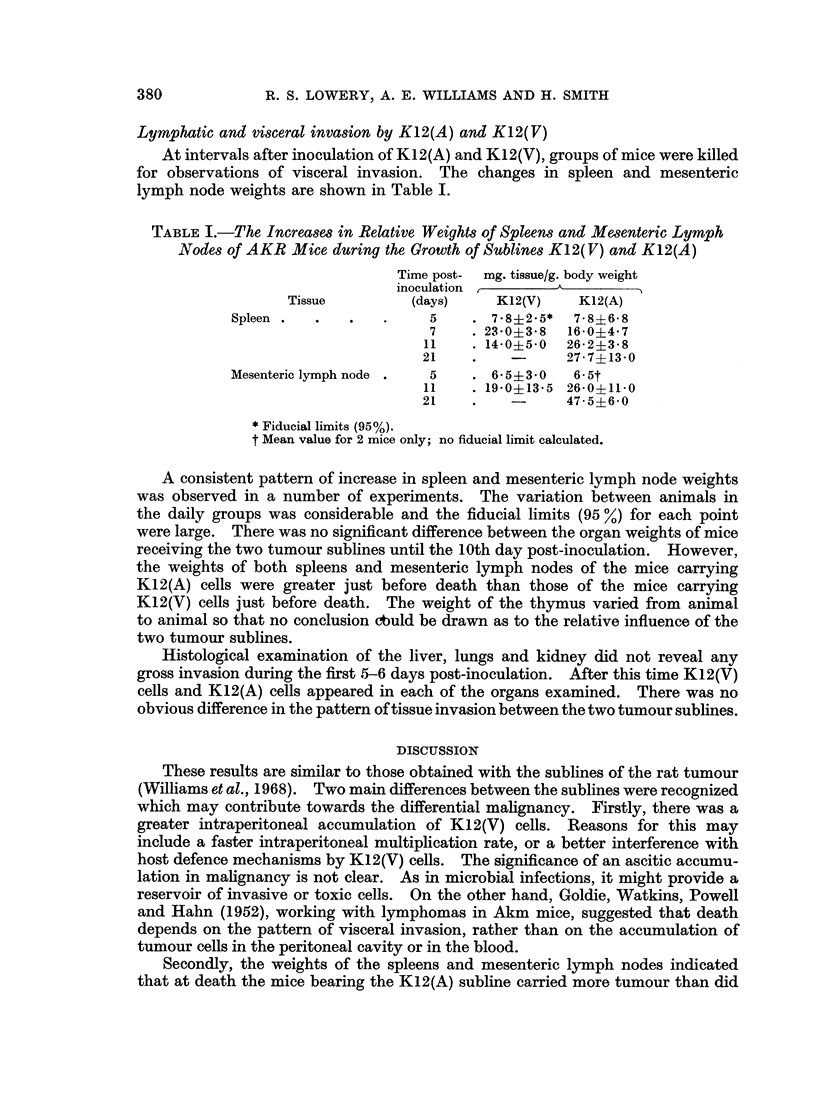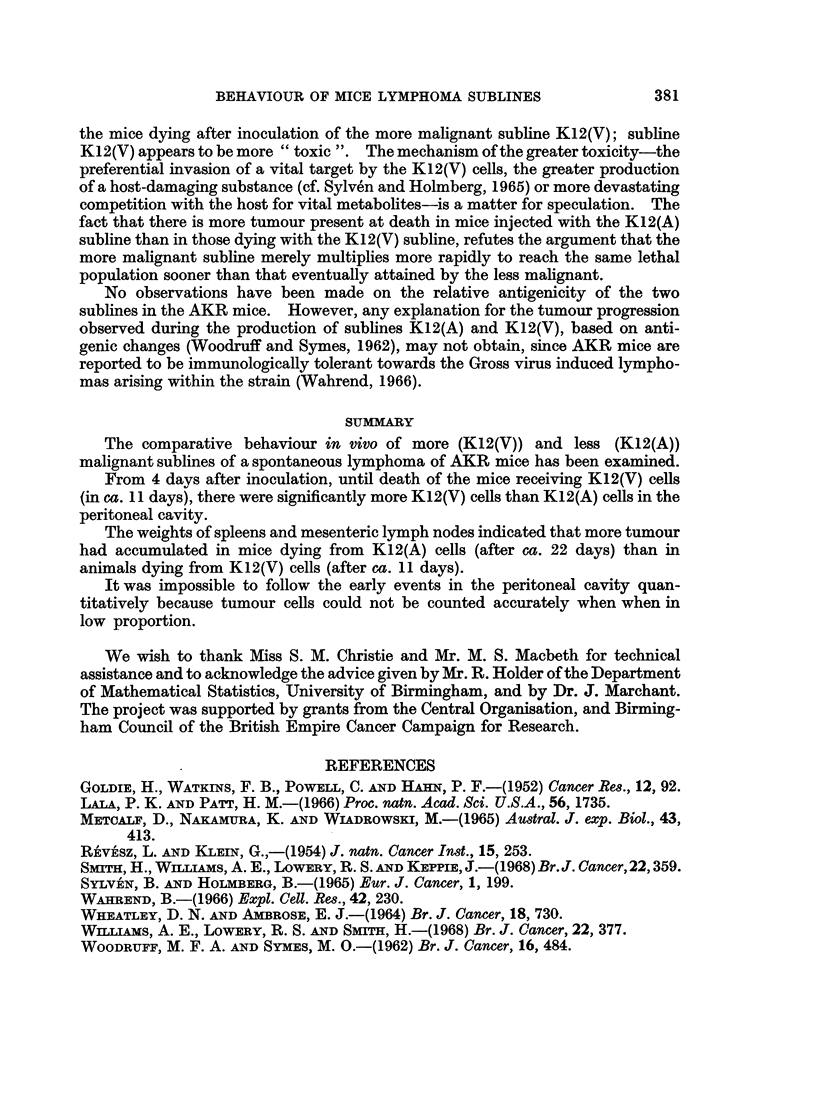# The behaviour in AKR mice of lymphoma sublines of low and high malignancy.

**DOI:** 10.1038/bjc.1968.46

**Published:** 1968-06

**Authors:** R. S. Lowery, A. E. Williams, H. Smith


### THE BEHAVIOUR IN AKR MICE OF LYMPHOMA SUBLINES OF LOW AND HIGH MALIGNANCY

R. S. LOWERY*, A. E. WILLIAMS AND H. SMITH

*From the Department of Microbiology, The University of Birmingham, Birmingham, 15*

Received for publication April 1, 1968

SMITH, Williams, Lowery and Keppie (1968) described the production of two sublines of an AKR mouse lymphoma (K12(A) and K12(V)) with differing malignancy. The sublines were derived by tumour progression during passage of a newly isolated spontaneous lymphoma (K12). The cells of the 6th passage constituted the subline of low malignancy (K12(A)) and those of the 14th passage constituted the subline of high malignancy (K12(V)).

This paper describes studies of the intraperitoneal growth and the blood and visceral invasion of the two sublines. Such studies have been made with individual mouse tumours (Révész and Klein, 1954; Wheatley and Ambrose, 1964; Lala and Patt, 1966) but not, as far as the authors are aware, with pairs of closely related sublines of differing malignancy.

### MATERIALS AND METHODS

### *Mice*

These were inbred AKR mice (20–24 g.), bred under specific pathogen-free conditions. The isogenicity of the animals was checked at intervals by skin grafting. Both male and female mice were used, as no significant difference was observed between the mean death times produced in male or female mice (Smith *et al.* 1968).

### *Tumour sublines K12(A) and K12(V)*

Derivation and storage of these sublines was described by Smith *et al.*, (1968). With $1 \times 10^6$ viable cells given intraperitoneally, the mean death time of mice (20–24 g.) injected with K12(A) cells was 25·1 days; that of mice receiving K12(V) cells was 11·6 days.

### *Inoculation of tumour sublines*

In comparative studies standard doses ($1 \times 10^6$ viable cells in 0·1 ml.) of the two sublines were inoculated into randomized groups of mice.

### *Estimation of the number of tumour cells in the peritoneal cavity and blood of mice at intervals after injection of the tumour sublines*

At intervals over 14 days, animals were killed by cervical dislocation and the numbers of free tumour cells in the peritoneal cavity and blood were estimated.

* Present address: Department of Botany and Zoology, Sir John Cass College, Jewry Street, London, E.C.3.

*Peritoneal cavity.*—The total number of free host and tumour cells was determined by the dye-dilution technique of Révész and Klein (1954). Differential counts were done on smears of intraperitoneal cells in calf serum (cells were centrifuged from the suspension used for total counts) stained by the Giemsa technique of Metcalf, Nakamura and Wiadrowski (1965). In differential counts, cells were classed as " tumour " or "host "; no further subdivision was attempted. Primitive lymphoid cells of the type described by Metcalf *et al.*, (1965) were " tumour ". Although lymphoma cells could be distinguished from most host cells, some confusion between then and the normal lymphoid series was inevitable and a source of error, particularly at low lymphoma cell concentrations (see below).

Differential counts were performed in ignorance of the identity of the sample; 1000–1500 cells were counted in several bands across the three smears on each slide. In all experiments duplicate differential counts were made on a proportion of the slides. If the ratio greater tumour percentage/lesser tumour percentage was $> 1 \cdot 5$ the sample was rejected. In preliminary work, 40–60 per cent of samples selected for recount, taken from groups of animals during the first 3 days post-inoculation, were rejected and experiments during this period were abandoned. The failure to obtain reproducible results during days 1–3 was due to the sparsity of tumour cells in the smears. As the proportion of tumour cells increased, the tumour cell counts of the peritoneal sample became more reproducible and from 4 days after inoculation were sufficiently consistent for comparative purposes. In several experiments, the ratio greater tumour percentage/ lesser tumour percentage was within the accepted limit ($\ngtr 1 \cdot 5$) for 80 per cent of the duplicate counts (cf. Table I; Williams, Lowery and Smith, 1968). In some experiments only 25 per cent of the samples were counted in duplicate and any errors which occurred in the remaining 75 per cent were accounted for in the variation of the mean tumour count of the whole group of animals.

*Blood.*—The leucocyte concentration in heart blood was measured by haemocytometer. Differential counts of " tumour " and " host " cells were done as described above. After the 6th day following inoculation, duplicate differential counts, made on one out of the four daily samples, were reproducible within the limits laid down for intraperitoneal smears.

*Weight and histological examinations of tissues invaded by the tumour sublines*

The wet weights of the thymus, the mesenteric lymph node and the spleen and of the mouse were obtained and the ratio weight of organ (mg.)/weight of mouse(g.) was calculated. Organs for histological examination were fixed in formal saline or Bouin and sections were stained with haematoxylin and eosin.

### RESULTS

*Comparison of the increases in tumour cell population of sublines K12(A) and K12(V) within the peritoneal cavity*

The two sublines were injected intraperitoneally and on each day following the 3rd day after inoculation 2–4 mice were killed for determination of the number of free tumour cells within the peritoneal cavity.

Fig. 1 shows the changes in free tumour cell populations of the two sublines from the pooled data of two experiments. After the 6th day post-inoculation, the cell population of the more malignant subline K12(V) was significantly higher than

FIG. 1.—The changes in intraperitoneal tumour cell populations of sublines K12(A)( △; fiducial limits (95%)⫫) and K12(V) (○; fiducial limits (95%) =). The data is derived from two similar comparative experiments (one involving the examination of 2 mice per day and the other 3 mice per day) representative of 5 experiments.

(i) Where no lower fiducial limit is shown, this indicates a wide spread of values for individual samples.

(ii) On day 5, smears derived from 3 of the 5 mice receiving K12(V) in the 2 experiments were poor; consequently, the relevant point is based on the mean of 2 samples counted (one from each experiment).

that of the less malignant subline, a difference maintained until the death of the mice with K12(V). The cell population of K12(A) decreased from day 7 to day 11 and did not return to a higher level during the remaining life of the mice.

*Invasion of the blood*

Although reproducible counts of tumour cells were obtained on blood samples taken from day 7, large variation in the tumour cell counts between individual mice on the same day prevented any definite conclusions being drawn about differences between the two sublines. However, the tumour cell concentration of both sublines appeared to increase similarly until the death of those mice carrying K12(V). This was followed by a tendency for a further increase of the tumour cell concentration in the blood of the mice carrying K12(A).

*Host cell changes during growth of sublines K12(A) and K12(V)*

Host cell counts in the peritoneal cavity and blood indicated an inflammatory response parallelling the increase in tumour cell numbers. This response was not significantly different in magnitude whichever subline had been injected.

*Lymphatic and visceral invasion by K12(A) and K12(V)*

At intervals after inoculation of K12(A) and K12(V), groups of mice were killed for observations of visceral invasion. The changes in spleen and mesenteric lymph node weights are shown in Table I.

TABLE I.—*The Increases in Relative Weights of Spleens and Mesenteric Lymph Nodes of AKR Mice during the Growth of Sublines K12(V) and K12(A)*

| Tissue | Time post-inoculation (days) | mg. tissue/g. body weight K12(V) | K12(A) |
|---|---|---|---|
| Spleen . . . . | 5 | . 7·8±2·5* | 7·8±6·8 |
| | 7 | . 23·0±3·8 | 16·0±4·7 |
| | 11 | . 14·0±5·0 | 26·2±3·8 |
| | 21 | . — | 27·7±13·0 |
| Mesenteric lymph node . | 5 | . 6·5±3·0 | 6·5† |
| | 11 | . 19·0±13·5 | 26·0±11·0 |
| | 21 | . — | 47·5±6·0 |

* Fiducial limits (95%).
† Mean value for 2 mice only; no fiducial limit calculated.

A consistent pattern of increase in spleen and mesenteric lymph node weights was observed in a number of experiments. The variation between animals in the daily groups was considerable and the fiducial limits (95 %) for each point were large. There was no significant difference between the organ weights of mice receiving the two tumour sublines until the 10th day post-inoculation. However, the weights of both spleens and mesenteric lymph nodes of the mice carrying K12(A) cells were greater just before death than those of the mice carrying K12(V) cells just before death. The weight of the thymus varied from animal to animal so that no conclusion could be drawn as to the relative influence of the two tumour sublines.

Histological examination of the liver, lungs and kidney did not reveal any gross invasion during the first 5–6 days post-inoculation. After this time K12(V) cells and K12(A) cells appeared in each of the organs examined. There was no obvious difference in the pattern of tissue invasion between the two tumour sublines.

### DISCUSSION

These results are similar to those obtained with the sublines of the rat tumour (Williams *et al.*, 1968). Two main differences between the sublines were recognized which may contribute towards the differential malignancy. Firstly, there was a greater intraperitoneal accumulation of K12(V) cells. Reasons for this may include a faster intraperitoneal multiplication rate, or a better interference with host defence mechanisms by K12(V) cells. The significance of an ascitic accumulation in malignancy is not clear. As in microbial infections, it might provide a reservoir of invasive or toxic cells. On the other hand, Goldie, Watkins, Powell and Hahn (1952), working with lymphomas in Akm mice, suggested that death depends on the pattern of visceral invasion, rather than on the accumulation of tumour cells in the peritoneal cavity or in the blood.

Secondly, the weights of the spleens and mesenteric lymph nodes indicated that at death the mice bearing the K12(A) subline carried more tumour than did the mice dying after inoculation of the more malignant subline K12(V); subline K12(V) appears to be more " toxic ". The mechanism of the greater toxicity—the preferential invasion of a vital target by the K12(V) cells, the greater production of a host-damaging substance (cf. Sylvén and Holmberg, 1965) or more devastating competition with the host for vital metabolites—is a matter for speculation. The fact that there is more tumour present at death in mice injected with the K12(A) subline than in those dying with the K12(V) subline, refutes the argument that the more malignant subline merely multiplies more rapidly to reach the same lethal population sooner than that eventually attained by the less malignant.

No observations have been made on the relative antigenicity of the two sublines in the AKR mice. However, any explanation for the tumour progression observed during the production of sublines K12(A) and K12(V), based on antigenic changes (Woodruff and Symes, 1962), may not obtain, since AKR mice are reported to be immunologically tolerant towards the Gross virus induced lymphomas arising within the strain (Wahrend, 1966).

SUMMARY

The comparative behaviour *in vivo* of more (K12(V)) and less (K12(A)) malignant sublines of a spontaneous lymphoma of AKR mice has been examined.

From 4 days after inoculation, until death of the mice receiving K12(V) cells (in *ca.* 11 days), there were significantly more K12(V) cells than K12(A) cells in the peritoneal cavity.

The weights of spleens and mesenteric lymph nodes indicated that more tumour had accumulated in mice dying from K12(A) cells (after *ca.* 22 days) than in animals dying from K12(V) cells (after *ca.* 11 days).

It was impossible to follow the early events in the peritoneal cavity quantitatively because tumour cells could not be counted accurately when when in low proportion.

We wish to thank Miss S. M. Christie and Mr. M. S. Macbeth for technical assistance and to acknowledge the advice given by Mr. R. Holder of the Department of Mathematical Statistics, University of Birmingham, and by Dr. J. Marchant. The project was supported by grants from the Central Organisation, and Birmingham Council of the British Empire Cancer Campaign for Research.

### REFERENCES

GOLDIE, H., WATKINS, F. B., POWELL, C. AND HAHN, P. F.—(1952) *Cancer Res.*, **12**, 92.
LALA, P. K. AND PATT, H. M.—(1966) *Proc. natn. Acad. Sci. U.S.A.*, **56**, 1735.
METCALF, D., NAKAMURA, K. AND WIADROWSKI, M.—(1965) *Austral. J. exp. Biol.*, **43**, 413.
RÉVÉSZ, L. AND KLEIN, G.,—(1954) *J. natn. Cancer Inst.*, **15**, 253.
SMITH, H., WILLIAMS, A. E., LOWERY, R. S. AND KEPPIE, J.—(1968) *Br. J. Cancer*, **22**, 359.
SYLVÉN, B. AND HOLMBERG, B.—(1965) *Eur. J. Cancer*, **1**, 199.
WAHREND, B.—(1966) *Expl. Cell. Res.*, **42**, 230.
WHEATLEY, D. N. AND AMBROSE, E. J.—(1964) *Br. J. Cancer*, **18**, 730.
WILLIAMS, A. E., LOWERY, R. S. AND SMITH, H.—(1968) *Br. J. Cancer*, **22**, 377.
WOODRUFF, M. F. A. AND SYMES, M. O.—(1962) *Br. J. Cancer*, **16**, 484.